# The immediate effect of arch support insoles on the biomechanics of the lower limbs during the forward lunge step in badminton

**DOI:** 10.7717/peerj.21113

**Published:** 2026-04-14

**Authors:** Xingyu Wu, Huiting Liang, Chuxian He, Nachiappan Chockalingam, Renjie Li, Zhonghao Xie, Bosi Chen, Bowei Wang, Zhiguan Huang

**Affiliations:** 1 School of Sports and Health, Guangzhou Sport University, Guangzhou, Guangdong, China; 2 School of National Badminton, Guangzhou Sport University, Guangzhou, Guangdong, China; 3 Guangdong Vocational Academy of Art, Guangzhou, Guangdong, China; 4 Faculty of Health Sciences, Staffordshire University, Stoke-on-Trent, United Kingdom; 5 School of Physical Education, Guangzhou Sport University, Guangzhou, Guangdong, China; 6 Foshan Linzhi Polymer Materials Science And Technology Co. Ltd, Foshan, Guangdong, China; 7 Guangdong Engineering Technology Research Center for Sports Aids, Guangzhou Sport University, Guangzhou, Guangdong, China

**Keywords:** Arch support insoles, Patellofemoral joint load, Forward lunges, Lower limb biomechanics

## Abstract

**Background:**

The lunge step in badminton involves frequent impacts that contribute to lower-limb injuries. Although arch support insoles (ASI) are widely used for sports protection, studies on their application in badminton are limited, and internal loading during the lunge maneuver has rarely been examined. This study aimed to investigate the immediate effects of functional arch support insoles on lower-limb biomechanics and patellofemoral joint loading during a forward lunge in badminton.

**Methods:**

Sixteen students from a badminton class at the University of Physical Education performed a forward lunge using both original and arch support insoles. Kinematic and kinetic data were collected using a motion capture system and a force platform, while patellofemoral joint loading was estimated *via* a mathematical model based on cadaveric data.

**Results:**

Compared with the original insole, the arch support insole results in a smaller knee external rotation angle (*P* = 0.030, ES = 0.60), a larger ankle dorsiflexion angle at initial contact (*P* = 0.024, ES = 0.63), and a peak knee external rotation angle during the phase (*P* = 0.042, ES = 0.55); they also produce a reduced vertical ground reaction force loading rate (*P* = 0.003, ES = 0.90) and peak negative ankle power (*P* = 0.026, ES = 0.62). Additionally, peak patellofemoral joint stress (*P* = 0.006, ES = 0.81) and stress-time integral (*P* = 0.019, ES = 0.66) were significantly increased.

**Conclusion:**

The results indicate that ASI altered ankle cushioning strategies, producing lower vertical ground reaction force loading rates. Furthermore, changes in hip and knee metrics suggest a potential reduction in dynamic knee valgus patterns. These results suggest that ASI may be able to reduce injury risk in badminton. However, ASI may also increase patellofemoral joint loading due to greater knee extension moments. Thus, balancing injury prevention and potential risks is crucial. Future research should investigate individualized fit strategies and the long-term biomechanical effects of ASI.

## Introduction

Badminton is one of the most popular racket sports among people worldwide (Valldecabres et al., 2020), and it has developed with a broad entertainment foundation and well-organized elite-level competitions around the world (Mei et al., 2017). Fast and efficient footwork is required for players to reach the shuttle and return to the court center for the next stroke (Phomsoupha & Laffaye, 2014). Among all footwork patterns, the lunge is particularly common, accounting for more than 15% of total movements (Hong et al., 2014). Badminton is characterized by high intensity and explosive actions (Kuntze, Mansfield & Sellers, 2010). Frequent and forceful impacts from repetitive lunges contribute to a high incidence of lower-limb injuries (Cronin, McNair & Marshall, 2003; Robinson & O’Donoghue, 2008; Kuntze, Mansfield & Sellers, 2010). Consequently, injury prevention remains a key concern for both professional athletes and recreational badminton enthusiasts.

Characteristics of shoes and insoles (*e.g.*, midsole hardness, thickness, torsional stiffness, arch support, materials, and shape) have been modified to reduce injury risk and enhance performance (Hoitz et al., 2020; Lam et al., 2022a). Foot-based biomechanical interventions have been used to prevent many lower limb musculoskeletal disorders, such as through footwear, insoles, and taping techniques (Kayll et al., 2023). For instance, a meta-analysis found that foot orthotics reduced the overall injury risk by 28% and the risk of lower limb stress fractures by 41%, whereas shock-absorbing insoles were not found to be effective in preventing injuries (Bonanno et al., 2017). Recently, many athletes have begun wearing functional arch support insoles (ASI) to enhance athletic performance and reduce injuries (Chen, Peng & Wei, 2022). Studies have demonstrated positive effects of ASI on lower-limb biomechanics compared with flat insoles during walking (Zhao et al., 2021), stair descent (Bonifácio et al., 2018), jogging (Zhao et al., 2021), archery (Wu et al., 2022), basketball (Lam et al., 2022b), and landing tasks (Jor et al., 2025). Specifically, arch support insoles reduce ankle valgus and knee internal rotation, lower vertical loading rates, and improve knee and ankle stability during sport tasks (Tillman et al., 2003; Wang et al., 2020). Previous authors have noted that the biomechanical changes brought about by ASI are favorable in response to deceleration, sharp stops, and directional changes in badminton. These changes may improve athletes’ movement efficiency on the court and reduce the occurrence of knee anterior cruciate ligament (ACL) injuries (Chen, Peng & Wei, 2022). However, research on the application of ASI in badminton remains limited. This study, therefore, aimed to examine the biomechanical effects of ASI on badminton lunge performance.

Lunge training and repetitive practice can lead to overuse injuries due to accumulated mechanical loading (Shariff, George & Ramlan, 2009). Previous studies have extensively examined lower-limb loading characteristics during the badminton lunge, showing that ground reaction force loading rates increase when the ankle dorsiflexor muscles are fatigued (Tong et al., 2023). Moreover, the inherently high-impact nature of the lunge has been identified as a potential contributor to lower-limb overuse injuries (Huang et al., 2025). Common knee joint injuries associated with this include patellofemoral pain syndrome and patellar tendinopathy. Pain or dysfunction in the anterior knee region may restrict flexion and extension, impairing normal lunge movements and reducing athletic performance. However, research on patellofemoral joint stress (PFJS) during badminton-specific lunge movements remains extremely limited. Based on our literature search, only one published study has estimated PFJS during the lunge in badminton (Yu et al., 2021), thereby highlighting a clear knowledge gap about PFJS in badminton sports biomechanics. Moreover, existing research on the influence of ASI on patellofemoral joint loading has primarily examined functional tasks such as running, walking, and stair descent, while sport-specific activities, particularly those related to badminton, have been largely overlooked (Hart et al., 2022; Kayll et al., 2023). Prior studies have highlighted that mathematical modeling approaches are among the most commonly used methods for estimating PFJS due to their computational efficiency and ease of input data acquisition (Wang et al., 2023). Therefore, the present study incorporates traditional biomechanical evaluation metrics alongside patellofemoral joint load estimations derived from a two-dimensional mathematical model. The objective is to comprehensively assess the effects of ASI on lower limb biomechanics during badminton lunges through a multidimensional set of indicators.

This study aimed to explore the immediate effects of functional arch support insoles on lower limb kinematics, kinetics, and patellofemoral joint loading during the forward lunge step in badminton. Specifically, we sought to investigate: (1) whether functional arch support insoles, compared to original insoles, may influence lower limb kinematics and impact loads; and (2) whether they are associated with changes in patellofemoral joint stress during badminton lunges.

## Materials and Methods

### Subjects

Sixteen male participants were recruited from the badminton specialization class at Guangzhou Sport University representing a convenience sample of trained university-level badminton players. Given the limited number of female athletes (*n* = 3) enrolled in the badminton specialization class, and to ensure a homogenous sample for controlled biomechanical analysis, only male participants were included in this study. Their mean age was 24.20 ± 1.40 years, height 1.76 ± 0.05 m, body mass 69.57 ± 9.10 kg, and BMI 22.47 ± 2.25 kg/m^2^. Inclusion criteria were: (1) no injuries in the lower limbs and feet within 1 year; (2) foot test results for healthy feet; (3) right-handed racket dominance; and (4) a training history of at least 3 years and more than 5 years of badminton playing experience. Exclusion criteria were: (1) any musculoskeletal disorders or surgeries in the lower limbs or feet within the past 12 months; (2) presence of foot deformities or flatfoot; (3) left-handed racket dominance; and (4) beginners or athletes with less than 3 years of systematic training. All subjects provided written informed consent in accordance with the protocol (NO. 2022LCLL-12) approved by the Ethics Review Committee of Guangzhou Sport University. The sample size (*n* = 16) was consistent with previous sport biomechanics studies employing paired designs and motion capture methods (Herbaut & Delannoy, 2020; Chen, Peng & Wei, 2022; Tong et al., 2023). Given the exploratory nature of this study, no formal a priori power analysis was performed.

### Testing shoes and insoles

**Figure 1:**
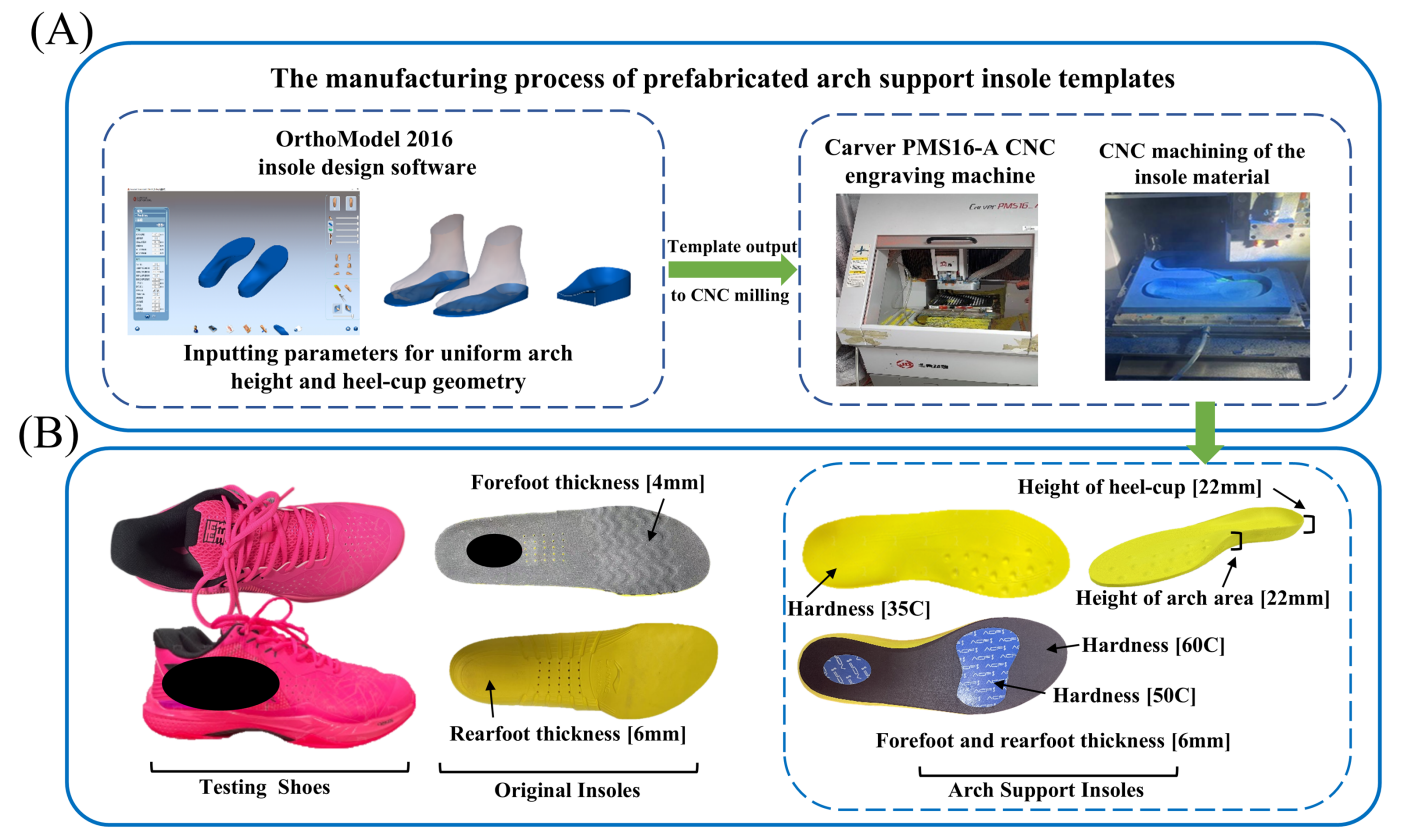
Details of the testing shoes, insoles, and the manufacturing process of the prefabricated arch support insoles. (A) Manufacturing process of the prefabricated arch support insole templates. Standardized templates for different shoe sizes were designed in OrthoModel 2016 software by inputting uniform geometric parameters (*e.g.*, arch height and heel-cup geometry). The resulting design templates were then output for CNC milling and fabricated using a Carver PMS16-A CNC engraving machine. (B) Images of the testing shoes, original insoles, and final arch support insoles. The dimensions and material hardness of the insoles are specified, including forefoot thickness (4 mm), rearfoot thickness (6 mm), arch area height (22 mm), and heel-cup height (22 mm). Hardness values are indicated for the respective areas of the insole (35C, 50C, 60C). Abbreviations: C refers to the Shore hardness scale.

All participants wore the same commercially available badminton shoe model, and standardized new badminton socks were provided to ensure consistency across trials. The control insoles used the original EVA insole provided with the shoes (Fig. 1B). The experimental insoles were the prefabricated arch support insoles. The ASIs for different shoe sizes were designed as templates in OrthoModel 2016 and machined using a Carver PMS16-A CNC machine, featuring standardized geometric parameters. Each participant used the ASI template that corresponded to their shoe size (ranging from 26 to 28 cm). Besides the standardized medial arch height and heel-cup height, the ASIs incorporated localized cushioning layers beneath the metatarsal and calcaneal regions. The workflow for producing the prefabricated ASIs and the detailed structural specifications of the insoles are presented in Figs. 1A and 1B. The entire design and manufacturing process was completed at the Guangdong Engineering Research Center of Sports Auxiliary Appliances.

### Data collection

**Figure 2:**
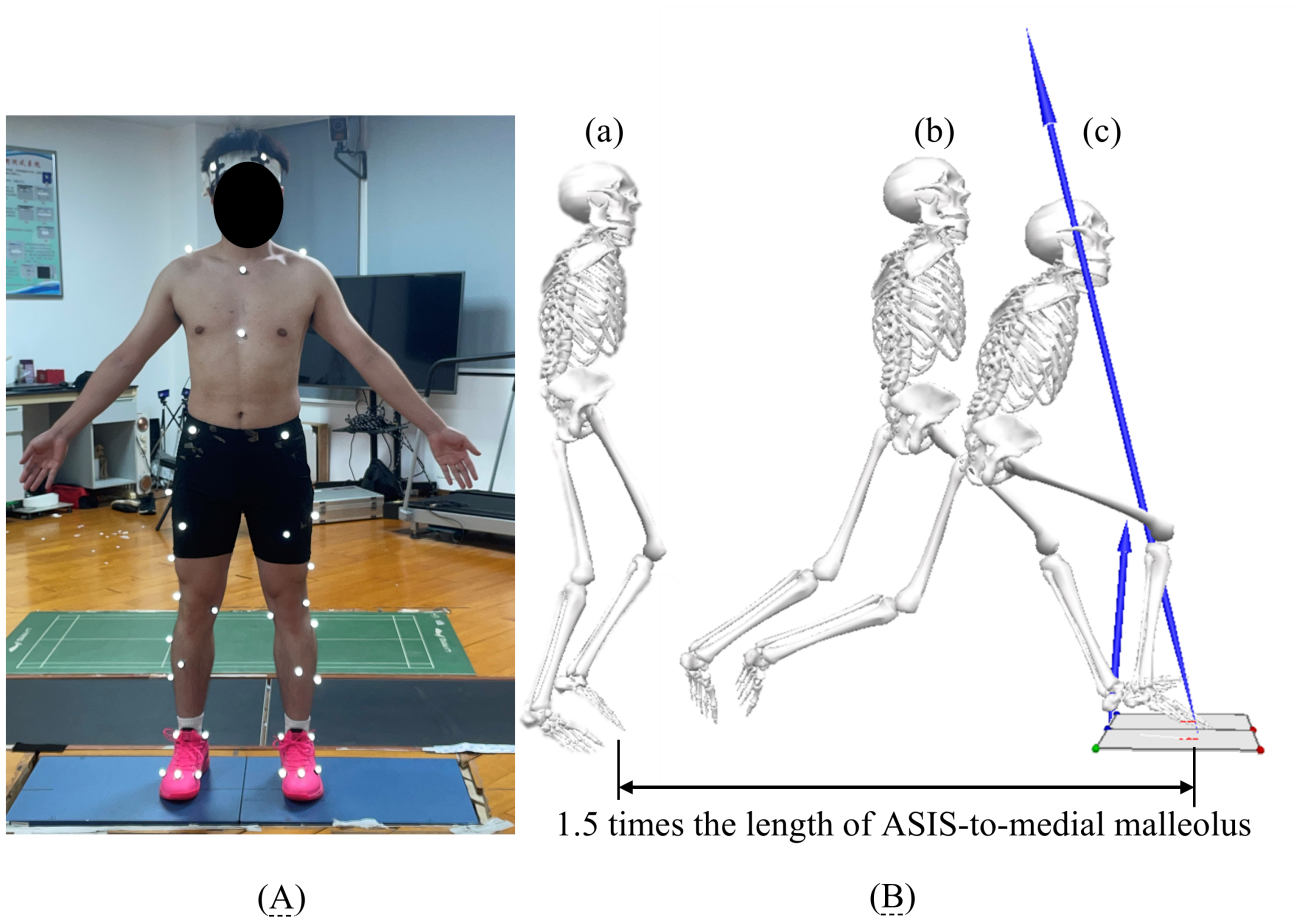
Static model and forward lunge movement breakdown. (A) Static model used for motion capture, (B) Forward lunge movement breakdown: (a) starting point, (b) initial contact moment upon landing, and (c) the peak moment of knee flexion angle. The distance from the anterior superior iliac spine (ASIS) to the medial malleolus is marked at 1.5 times the participant’s leg length to standardize lunge distance. Abbreviation: *ASIS* refers to the anterior superior iliac spine, a bony prominence on the pelvis used as a reference point for biomechanical measurements.

The experiment was conducted in the Sports Biomechanics Laboratory of Guangdong Sports Aids Engineering Experimental Center. Participants were instructed to avoid strenuous activity for 24 h before testing. The testing procedures and safety measures were explained in advance. After subjects changed into tights for stretching and warming up, reflect markers (diameter 12 mm) were attached to anatomical landmarks. The static model is presented in Fig. 2A. The anterior superior iliac spine (ASIS) to medial malleolus length was measured with a tape to standardize lunge distance. Each subject’s starting position (as shown in Fig. 2B) was 1.5 times their leg length from the center of the force platform (Kuntze, Mansfield & Sellers, 2010). The starting point of the lunge was marked with tape, and subjects subsequently performed adaptive forward lunge maneuvers based on their individual lunge lengths, completing at least five correct forward stance trials for familiarization. Prior to these practice trials, all participants received standardized verbal instructions and a visual demonstration emphasizing that the lunge should be performed with maximum effort, simulating match-play intensity. Subjects wore the two types of insole in a random order. The testing sequence was manually randomized by the experimenter using a simple random allocation method to eliminate any potential order effects and learning-related bias. Motion data were recorded using an eight-camera Vicon system (200 Hz; Oxford Metrics, Oxford, UK) synchronized with two embedded AMTI force plates (1,000 Hz; Advanced Mechanical Technology, Inc., Watertown, MA, USA).

Participants initiated the forward lunge from the marked start point with maximal effort, stepping onto the force plate with the right foot. One-minute rest intervals were provided between trials to minimize fatigue. Three valid trials per insole condition were averaged for analysis.

### Data processing

Marker trajectories were manually identified in the Vicon software. Spline interpolation was used for the missing data points, with three frames before and after (Wang et al., 2020). Data were exported to Visual 3D (C-Motion Inc, Germantown, MD, USA) for further analysis. Kinematic and kinetic data were filtered with a fourth-order low-pass Butterworth filter at 15 and 25 Hz (Hanzlíková et al., 2016). The joint moments were calculated using standard inverse kinetic methods. The vertical ground reaction force (vGRF) was normalized to body weight (Kuntze, Mansfield & Sellers, 2010). The cushioning phase was defined from initial contact to the time of maximum knee flexion angle (as shown in Figs. 2B(b) and 2B(c)), where initial contact was identified as the instant when the vGRF first exceeded 15 N. The vertical loading rate is the average slope of the vGRF curve from initial contact to the time of the first peak vGRF, covering 0%–100% of the phase (Lam et al., 2018; Tong et al., 2023) Eq. (1). The impulse was calculated as the time integral of the force curve during the cushioning phase Eq. (2) (Nielsen et al., 2020; Huang et al., 2025). Joint angles followed the right-hand rule: positive values indicate hip flexion/adduction/internal rotation, knee flexion/varus/internal rotation, and ankle dorsiflexion/inversion/adduction.

(1) \begin{eqnarray*}VLR& = \frac{{F}_{IC}-{F}_{FPvGRF}}{{t}_{IC}-{t}_{FPvGRF}} \end{eqnarray*}

(2) \begin{eqnarray*}Impulse& =\int \nolimits \nolimits _{{t}_{MKF}}^{{t}_{IC}}F(t)~dt\end{eqnarray*}

where VLR is the loading rate of vGRF (BW/s); *F*_IC_ is the vGRF at the instant of initial contact (BW); *F*_FPvGRF_ is the first peak value of vGRF (BW); t_IC_ is the time at initial contact (s); t_FPvGRF_ is the time at the first peak vGRF (s); and t_MKF_ is the time at maximum knee flexion (s).

The present study employed a two-dimensional mathematical model to calculate the load on the patellofemoral joint (Van Eijden et al., 1986; Nunes et al., 2018). Although previous attempts have been made to improve the accuracy of PFJS estimation using musculoskeletal modeling, finite element analysis (FEA), and discrete element analysis (DEA), the mathematical analytical model remains the most commonly used method for estimating PFJS due to its simple computational process and the ease of obtaining the necessary input variables. This analytical model has been widely applied to estimate PFJS during dynamic tasks such as running, forward lunge exercise, depth jumping, and barbell back squatting. However, this method also has limitations, such as neglecting the co-contraction of knee flexor muscles and considering only the sagittal-plane motion of the knee joint, so caution should be exercised when interpreting the results of this study. All PFJS-related formulas were implemented using custom expressions through the “Evaluate Expression” pipeline function in Visual3D (C-Motion, Inc.). The specific formulae are as follows:

(3) \begin{eqnarray*}L{A}_{eff} \left( {X}_{i} \right) & =8\cdot 1{0}^{ \left( -8 \right) }{ \left( {X}_{i} \right) }^{3}-1.29\cdot 1{0}^{ \left( -5 \right) }{ \left( {X}_{i} \right) }^{2}+2.8\cdot 1{0}^{ \left( -4 \right) } \left( {X}_{i} \right) +0.046\end{eqnarray*}

(4) \begin{eqnarray*}{F}_{Q} \left( {X}_{i} \right) & ={M}_{EXT} \left( {X}_{i} \right) /{L}_{A} \left( {X}_{i} \right) \end{eqnarray*}

(5) \begin{eqnarray*}K& = \frac{-3.83\cdot 1{0}^{-5}{ \left( {X}_{i} \right) }^{2}+1.47\cdot 1{0}^{-3} \left( {X}_{i} \right) +0.462}{-6.98\cdot 1{0}^{-7}{ \left( {X}_{i} \right) }^{3}+1.55\cdot 1{0}^{-4}{ \left( {X}_{i} \right) }^{2}-0.0162 \left( {X}_{i} \right) +1} \end{eqnarray*}

(6) \begin{eqnarray*}PF{J}_{RF}& =k\cdot {F}_{Q}\end{eqnarray*}

(7) \begin{eqnarray*}{S}_{PFCA} \left( {X}_{i} \right) & =0.0781{ \left( {X}_{i} \right) }^{2}+0.6763 \left( {X}_{i} \right) +151.75\end{eqnarray*}

(8) \begin{eqnarray*}{S}_{PFJS} \left( {X}_{i} \right) & =PF{J}_{RF} \left( {X}_{i} \right) /{S}_{PFCA} \left( {X}_{i} \right) \end{eqnarray*}

(9) \begin{eqnarray*}{\overline{R}}_{PFJS}& =\Delta {S}_{PFJS}/\Delta t\end{eqnarray*}

(10) \begin{eqnarray*}{I}_{PFJS}& ={S}_{PFJS}~dt\end{eqnarray*}

where X_i_ is the knee flexion angle per frame (^∘^); M_EXT_ is the knee extension moment (Nm/kg); LA_eff_ is the quadriceps force arm (m); *F*_Q_ is the quadriceps muscle force (N/kg); PFJ_RF_ is the patellofemoral joint contact reaction force (N/kg); S_PFCA_ is the patellofemoral joint contact area (mm^2^); S_PFJS_ is the patellofemoral joint stress (MPa kg^−1^); ${\overline{\mathrm{R}}}_{\mathrm{PFJS}}$ is the average loading rate of patellofemoral joint stress (MPa (kg s)^−1^), and I_PFJS_ is the patellofemoral joint stress-time integral (MPas kg^−1^).

### Statistical analysis

All variables were expressed as mean ± standard deviation (SD), and the Shapiro–Wilk test demonstrated that the majority of the indicators exhibited a normal distribution. Consequently, paired-sample *t*-tests were employed for the indicators that followed a normal distribution, while Wilcoxon signed-rank tests were utilized for the indicators that did not meet normality. This approach was adopted to facilitate a comparative analysis of the impact of the cushioning performance of the insoles on the loading characteristics of the patellofemoral joints, as well as the parameters associated with the kinematics and kinetics of the lower limbs. Statistical significance was set at *α* = 0.05. The magnitude of the effect was determined by calculating the Cohen’s d, which was then classified as follows: *d* < 0.19 indicated a weak effect, *d* = 0.20 to 0.79 indicated a moderate effect and *d* ≥ 0.8 indicated a high effect. Because this study was exploratory, no multiple-comparison correction was applied. Effect sizes were provided to aid interpretation of practical relevance. All statistical analyses were conducted using SPSS version 26.0 (IBM Corporation, Armonk, NY, USA).

## Results

### The effect of ASI on lower limb kinematics

In terms of key kinematic changes, the use of ASI resulted in decreased knee external rotation and increased ankle dorsiflexion, both at initial contact and at peak during the cushioning phase.

**Figure 3:**
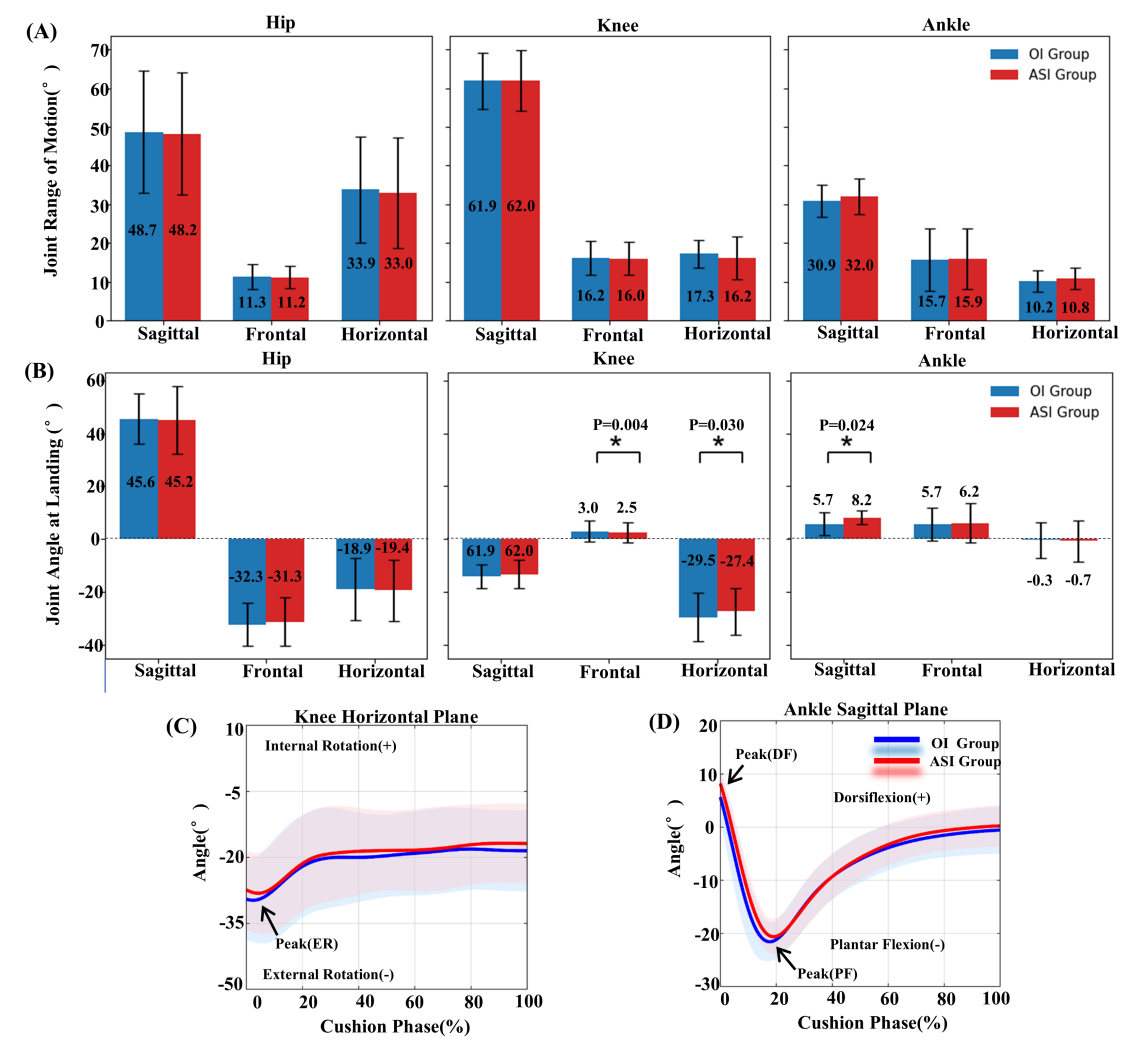
Joint range of motion and joint angle at initial contact with original insole (OI) and arch support insole (ASI) groups. (A) Joint range of motion during the landing cushion phase; (B) joint angle at initial contact. The numbers in the bars represent the average values of each joint angle (°) for the respective groups. Statistically significant differences are indicated by asterisks (*), with corresponding *p*-values provided. (C) Change in angle over time within the horizontal plane of the knee joint during the cushioning phase; (D) change in angle over time within the sagittal plane of the ankle joint during the cushioning phase. Images (C) and (D) are to visualize the peak joint angles in the landing cushion phase. Peak(ER), peak value of knee external rotation angle during cushion phase; PF(DF), peak value of ankle plantar flexion angle. These images are included to help readers better understand the timing of key movement events during the cushioning phase.

The joint angle results at initial contact are shown in Fig. 3B. Compared to the original insole (OI) group, the ASI group showed a significantly smaller knee external rotation angle (−27.39 ± 8.79° *vs.* −29.51 ± 9.15°, *P* = 0.030, ES = 0.60) at initial contact. The ASI group also had a significantly reduced knee varus angle (2.48 ± 3.87° *vs.* 3.00 ± 4.10°, *P* = 0.004, ES = 0.86). For the ankle joint, the ASI group showed significantly greater dorsiflexion at initial contact (8.23 ± 2.50° *vs.* 5.67 ± 4.30°, *P* = 0.024, ES = 0.63).

Peak values of joint angles during the cushioning phase are presented in Table 1, as shown in Figs. 3C and 3D. Peak knee external rotation angle was significantly lower in ASI group (−30.78 ± 9.04°) than OI (−32.68 ±  9.07°, *P* = 0.042, ES = 0.55). The peak ankle dorsiflexion angle was also significantly greater in the ASI group (9.56 ± 2.51°) compared to the OI group (7.18 ± 4.06°, *P* = 0.028, ES = 0.61).

**Table 1:** Peak joint angles during the landing cushion phase (values are mean ± SD).

		**Peak joint angle during cushion phase (^∘^)**		**T**	*p*-value	**ES**
		**OI Group**	**ASI Group**			
Hip	Flexion	93.58 ± 22.64	92.92 ± 26.50	0.398	0.696	0.10
	Abduction	−36.75 ± 10.63	−36.30 ± 11.89	−0.742	0.47	0.19
	Internal rotation	11.78 ± 18.61	11.22 ± 17.57	−0.317	0.755	0.08
	External rotation	−22.00 ± 15.36	−21.85 ± 14.54	−0.13	0.898	0.03
Knee	Flexion	−76.05 ± 8.03	−75.58 ± 9.11	−0.463	0.650	0.12
	Varus	17.72 ± 7.98	17.26 ± 8.17	0.667	0.515	0.17
	External rotation	−32.68 ± 9.07	−30.78 ± 9.04	−2.217	0.042^*^	0.55
Ankle	Dorsiflexion	7.18 ± 4.06	9.56 ± 2.51	−2.433	0.028^*^	0.61
	Plantar flexion	−23.68 ± 3.96	−22.43 ± 3.75	−1.518	0.150	0.38
	Inversion	8.45 ± 4.94	9.54 ± 5.67	−1.609	0.128	0.40
	Eversion	−7.22 ± 7.36	−6.35 ± 7.21	−0.934	0.365	0.23
	Internal rotation	4.50 ± 6.95	5.29 ± 7.88	−1.338	0.201	0.33
	External rotation	−6.35 ± 7.21	−5.76 ± 7.11	−0.443	0.664	0.11

**Notes.**

o Degree of angle.

* Significant difference between two groups.

ES, Effect size (Cohen’s d).

The description of the joint angle is consistent with the right-hand rule. A positive value for joint angle denotes hip flexion, adduction, and internal rotation; knee flexion, varus, and internal rotation; and ankle dorsiflexion, inversion, and adduction for respective orthogonal planes.

No statistically significant differences were observed between the two groups in other joint angles, including range of motion (as shown in Fig. 3A), flexion (dorsiflexion), extension, adduction, abduction, and internal or external rotation (*P* > 0.05).

### The effect of ASI on lower limb kinetics

In terms of key kinetic changes, the use of ASI resulted in increased hip abduction and knee extension moments, as well as decreased ankle negative power and vertical ground reaction force loading rate during the cushioning phase.

As shown in Tables 2 and 3, during the cushioning phase of the forward lunge, the ASI group showed a significantly greater peak hip abduction moment (−0.31 ± 0.65 Nm/kg) compared to the OI group (−0.03 ± 0.57 Nm/kg, *P* = 0.025, ES = 0.62). Additionally, the peak knee extension moment was significantly higher in the ASI group (1.43 ± 0.65 Nm/kg) than in the OI group (1.30 ± 0.63 Nm/kg, *P* = 0.004, ES = 0.84).

**Table 2:** Peak joint moments during the landing cushion phase (values are mean ± SD).

		**OI group**	**ASI group**	**T**	*p*-value	**ES**
	Peak Moment (Nm kg^−1^)				
Hip	Extension	−4.45 ± 1.50	−4.43 ± 1.44	−0.133	0.896	0.03
	Adduction	3.62 ± 1.01	3.65 ± 0.92	−0.276	0.786	0.07
	Abduction	−0.03 ± 0.57	−0.31 ± 0.65	2.494	0.025^*^	0.62
	Internal rotation	0.82 ± 0.54	0.87 ± 0.45	−0.481	0.637	0.12
	External rotation	−0.74 ± 0.49	−0.69 ± 0.43	−0.934	0.365	0.23
Knee	Flexion	−1.55 ± 0.55	−1.53 ± 0.61	−0.357	0.726	0.09
	Extension	1.30 ± 0.63	1.43 ± 0.65	−3.371	0.004^*^	0.84
	Adduction	0.52 ± 0.48	0.56 ± 0.43	−0.8	0.436	0.20
	Abduction	−0.69 ± 0.43	−0.72 ± 0.43	0.758	0.460	0.19
	Internal rotation	0.65 ± 0.36	0.60 ± 0.35	1.224	0.240	0.31
	External rotation	−0.15 ± 0.26	−0.17 ± 0.20	−0.404	0.692	0.10
Ankle	Dorsiflexion	0.93 ± 0.27	0.92 ± 0.26	0.202	0.843	0.05
	Plantar Flexion	−1.20 ± 0.47	−1.24 ± 0.40	0.921	0.371	0.23
	Inversion	0.20 ± 0.16	0.18 ± 0.15	1.68	0.114	0.42
	Eversion	−0.14 ± 0.15	−0.15 ± 0.14	1.311	0.209	0.33
	Internal rotation	0.13 ± 0.11	0.12 ± 0.09	1.236	0.235	0.31
	External rotation	−0.17 ± 0.25	−0.16 ± 0.21	−0.436	0.669	0.11
	vGRF Derivative Measures				
PvGRF (BW)		2.04 ± 0.38	2.07 ± 0.28	−0.727	0.479	0.10
VLR (BW/s)		49.43 ± 27.63	31.50 ± 17.11	3.591	0.003^*^	0.90
Impulse(BW s)		0.36 ± 0.036	0.35 ± 0.048	0.658	0.520	0.16

**Notes.**

* Significant difference between two groups.

ES Effect size (Cohen’s d) vGRF Vertical ground reaction force PvGRF peak value of vertical ground reaction force BW body weight VLR loading rate of vertical ground reaction force

A positive value for moment denotes hip flexion, adduction, and internal rotation; knee flexion, varus, and internal rotation; and ankle dorsiflexion, inversion, and adduction for respective orthogonal planes.

**Table 3:** Negative power and work during the cushioning phase (values are mean ± SD).

		**OI group**	**ASI group**	**T**	*p*-value	**ES**
Hip	Peak negative power (W/kg)	−44.6 ± 21.54	−40.6 ± 17.50	−1.584	0.143	0.39
	Negative work (J/kg)	−2.72 ± 1.39	−2.63 ± 1.36	−0.774	0.451	0.19
Knee	Peak negative power (W/kg)	−10.74 ± 7.36	−10.98 ± 7.15	0.324	0.751	0.08
	Negative work (J/kg)	−0.70 ± 0.55	−0.76 ± 0.54	1.735	0.103	0.43
Ankle	Peak negative power (W/kg)	−12.47 ± 3.89	−10.56 ± 3.09	−2.478	0.026^*^	0.62
	Negative work (J/kg)	−0.40 ± 0.14	−0.41 ± 0.13	0.359	0.725	0.09

**Notes.**

* Significant difference between two groups.

ES, Effect Size (Cohen’s d).

In contrast, the ASI group showed a significantly lower peak ankle negative power (−10.56 ± 3.09 W/kg) compared to the OI group (−12.47 ± 3.89 W/kg, *P* = 0.026, ES = 0.62). The vertical ground reaction force loading rate was also significantly lower in the ASI group (31.50 ± 17.11 N/(kg s)) than in the OI group (49.43 ± 27.63 N/(kg s), *P* = 0.003, ES = 0.90).

No statistically significant differences were observed between the two groups in other joint moments and power variables during the cushioning phase (*P* > 0.05).

### The effect of ASI on patellofemoral joint loading

In terms of key patellofemoral joint loading changes, the use of ASI resulted in increased peak patellofemoral joint stress and stress–time integral, accompanied by higher quadriceps muscle force and knee extension moment at the time of peak stress.

As shown in Table 4, the ASI group exhibited a significantly greater peak patellofemoral joint stress (0.0825 ± 0.0392 MPa kg^−1^) compared to the OI group (0.0736 ± 0.0365 MPa kg^−1^, *P* = 0.006, ES = 0.81). The stress–time integral of the patellofemoral joint was also significantly higher in the ASI group (0.0103 ± 0.007 MPa s kg^−1^) than in the OI group (0.0092 ± 0.0072 MPa s kg^−1^, *P* = 0.019, ES = 0.66).

**Table 4:** Result on Patellofemoral joint loading during cushion phase (values are mean ± SD).

**Patellofemoral joint stress**	**OI group**	**ASI group**	**T**	*p*-value	**ES**
Peak S_PFJS_/(MPa kg^−1^)	0.0736 ± 0.0365	0.0825 ± 0.0392	−3.23	0.006^*^	0.81
${\bar {\mathrm{R}}}_{\mathrm{PFJS}}$ (MPa (kg s)^−1^)	1.92 ± 1.00	1.91 ± 1.17	0.04	0.969	0.01
I_PFJS_ (MPas kg^−1^)	0.0092 ± 0.0072	0.0103 ± 0.007	−2.628	0.019^*^	0.66

**Notes.**

* Significant difference between two groups.

ES Effect size (Cohen’s d) Peak SPFJS peak value of patellofemoral joint stress RPFJS the average loading rate of patellofemoral joint stress IPFJS the patellofemoral joint stress-time integral

As shown in Table 5, at the time of peak patellofemoral joint stress, the quadriceps muscle force was significantly greater in the ASI group (42.00 ± 18.59 N kg^−1^) than in the OI group (39.04 ± 19.40 N kg^−1^, *P* = 0.046, ES = 0.55). The knee extension moment was also significantly higher in the ASI group (1.42 ± 0.65 Nm kg^−1^) compared to the OI group (1.28 ± 0.62 Nm kg^−1^, *P* = 0.005, ES = 0.83).

**Table 5:** Result on explanatory measure at the peak of patellofemoral joint stress (values are mean ± SD).

**Index**		**OI group**	**ASI group**	**T**	*p*-value	**ES**
Patellofemoral Joint Indicators	*F*_Q_ (N kg^−1^)	39.04 ± 19.40	42.00 ± 18.59	−2.177	0.046^*^	0.55
	LA_eff_ (m)	0.033 ± 0.003	0.033 ± 0.004	−0.197	0.847	0.05
	K	0.91 ± 0.029	0.91 ± 0.035	−0.086	0.933	0.02
	PFJ_RF_/(N kg^−1^)	35.65 ± 18.01	38.06 ± 17.07	−1.73	0.104	0.43
	S_PFCA_ (mm^2^ )	498.09 ± 102.31	482.41 ± 99.18	0.997	0.335	0.25
	vGRF (BW)	1.77 ± 0.24	1.77 ± 0.25	−0.035	0.927	0.01
Angle (^∘^)	Hip flexion	82.23 ± 22.27	80.36 ± 23.51	1.72	0.105	0.43
	Knee flexion	−61.36 ± 9.89	−59.58 ± 10.06	−1.091	0.292	0.27
	Ankle plantar flexion	−8.55 ± 5.59	−7.56 ± 4.46	−0.727	0.479	0.18
Moment (Nm kg^−1^)	Hip Extensor	−3.72 ± 1.46	−3.38 ± 1.25	−1.88	0.080	0.47
	Knee extensor	1.28 ± 0.62	1.42 ± 0.65	−3.332	0.005^*^	0.83
	Ankle plantar flexion	−0.49 ± 0.48	−0.43 ± 0.50	−0.727	0.479	0.18

**Notes.**

* significant different between two groups.

ES Effect size (Cohen’s d) FQ the quadriceps muscle force LAeff the quadriceps force arm PFJRF the patellofemoral joint contact reaction force SPFCA the patellofemoral joint contact area vGRF vertical ground reaction force ∘ degree of angle BW body weight

No statistically significant differences were found between the two groups in patellofemoral joint stress loading rate, quadriceps moment arm, coefficient K, patellofemoral joint pressure, contact area, triple-joint angles and moments, or vertical ground reaction force at the moment of peak stress (*P* > 0.05).

## Discussion

This study investigated the immediate effects of arch support insoles on lower-limb biomechanics and patellofemoral joint stress during the forward lunge in badminton. To achieve this, a multidimensional index system was established, incorporating kinematic, kinetic, and patellofemoral joint loading parameters. The results demonstrated that the ASI had a substantial impact on joint angles, joint moments, impact loads, and patellofemoral joint stress during the cushioning phase compared to the OI group. However, no significant difference was found in the peak vertical ground reaction force (PvGRF) between the two insole conditions.

### The effect of insoles on kinematics and kinetics

In the current study, the PvGRF in the ASI group was comparable to that in the OI group. However, the average loading rate of ground reaction force (VLR) and the peak negative ankle joint power were both significantly reduced. These findings support the first hypothesis of the study.

ASI can modify ankle kinematics, thereby altering the cushioning mechanism and reducing impact loading. High-impact landings are closely associated with overuse injuries in the lower limbs (Radin et al., 1973), and appropriate footwear design can mitigate the adverse mechanical responses during such movements (Lam, Ding & Qu, 2017; Lam et al., 2017; Pan et al., 2025). In the present study, the VLR was significantly lower in the ASI group than in the OI group. In previous badminton lunge research comparing different heel designs, elite athletes wearing rounded heel badminton shoes exhibited reductions in VLR that were consistent with the findings of the present study (Lam, Ding & Qu, 2017; Lam et al., 2017). This reduction indicates that ASI may help reduce the risk of overuse injuries during badminton lunges (Yang et al., 2024). Notably, ASI did not significantly alter PvGRF, which is consistent with findings from previous studies (Alirezaei Noghondar & Bressel, 2017; Chen, Peng & Wei, 2022). One proposed explanation for the observed reduction in impact loading is a greater range of joint motion—particularly increased dorsiflexion during early stance—which facilitates energy absorption through rotational motion (Clansey et al., 2014). During lunge cushioning, changes in ankle angle typically follow a sequence from peak dorsiflexion to toe flexion, and back to dorsiflexion (as shown in Fig. 3D).

In this study, the ASI group exhibited a significantly greater peak ankle dorsiflexion angle during the cushioning phase, as well as a higher dorsiflexion angle at initial contact. This suggests that the ankle joint underwent a more complete dorsiflexion-to-toe flexion transition, enabling more effective eccentric contraction of the ankle dorsiflexor muscles. Such changes may enhance impact energy absorption. Therefore, the observed modifications in ankle joint kinematics may help explain the reduction in the vertical ground reaction force (vGRF) loading rate. Previous studies have also indicated that variations in sagittal foot landing angles can lead to altered muscle control strategies (Lam, Ding & Qu, 2017; Lam et al., 2017). Regarding changes in ankle negative power, the present study found that although the negative power–time integral remained unchanged in the ASI group, the peak negative power was significantly reduced. This suggests that while the total energy absorbed by the ankle joint did not change, it was absorbed more gradually instead of abruptly. This altered cushioning mechanism aligns with the observed reduction in loading rate of vGRF.

ASI modified hip landing mechanics and knee kinematics in ways that are associated with known biomechanical factors relevant to ACL loading, without implying direct effects on injury risk. Previous studies have reported that ACL injuries are commonly associated with excessive knee valgus and external rotation (Shimokochi & Shultz, 2008). Dynamic knee valgus (DKV), which involves potentially hazardous movement patterns such as hip adduction, internal rotation, knee abduction, tibial external rotation, and ankle valgus (Weiss & Whatman, 2015), which is considered a key risk factor for ACL injuries (Zhou et al., 2017). In the present study, the ASI group demonstrated a reduction in knee external rotation angle at initial contact, a decrease in peak knee external rotation angle during the cushioning phase, and an increase in peak hip abduction moment. These findings suggest that ASI-induced changes in landing mechanics may reduce relative internal femoral rotation with respect to the tibia. Although direct experimental evidence linking gluteus medius activation and hip abduction moment during badminton lunges is not yet available, evidence from single-leg landing tasks indicates that diminished hip abductor activity is accompanied by reductions in hip abduction moment and impaired frontal-plane control (Patrek et al., 2011). In inverse dynamics, the net joint moment represents the internal torque generated by muscles, passive tissues, and joint structures to counteract the external moments produced by ground reaction forces and inertia (Winter, 2009). Therefore, the increased peak hip abduction moment observed in the ASI group likely reflects greater mechanical demand on the hip abductors. This higher mechanical demand may enhance their neural activation, thereby contributing to improved frontal-plane stability and reducing dynamic knee valgus during lunge landings.

Moreover, enhanced hip abductor activation may contribute to hip abduction, thereby counteracting the development of DKV and potentially lowering the risk of ACL injury.

### The effect of ASI on patellofemoral joint loading

The results of this study showed increased peak patellofemoral joint stress, patellofemoral joint stress-time integral, quadriceps muscle strength, and knee extension moment at peak stress in the ASI group compared to the OI condition. The results did not support Hypothesis 2 of this study.

Wearing ASI led to increased patellofemoral joint loading compared to the OI. The primary contributor to this increase was the elevated knee extension moment; however, it remains unclear whether these biomechanical changes are associated with the onset of clinical symptoms (Kayll et al., 2023). The present study found that peak patellofemoral joint stress was more than 12% higher in the ASI group than in the OI group. This increase was likely driven by a rise in patellofemoral joint contact force or a reduction in contact area (Lin et al., 2023). At the moment of peak patellofemoral joint stress, the patellofemoral joint contact reaction force (PFJ_RF_) exhibited an increasing trend, while the contact area showed no significant difference between the two conditions. Therefore, the increase in peak patellofemoral joint stress observed in this study may be attributable to a greater contact force. Similar conclusions have been reported in previous research (Ho, Blanchette & Powers, 2012). The quadriceps tendon and the patellar tendon function synergistically to generate compressive forces at the patella; accordingly, patellofemoral joint contact force is positively correlated with quadriceps muscle force. In the present study, quadriceps force (*F*_Q_) was significantly higher in the ASI group than in the OI group, aligning with the observed increase in PFJ_RF_. Net joint moments were calculated *via* inverse dynamics and divided by the quadriceps lever arm to estimate quadriceps muscle force. Theoretically, an increase in knee extension moment or a decrease in the quadriceps moment arm would result in elevated quadriceps force. However, at peak stress, the quadriceps moment arm did not differ significantly between groups. However, the ASI group demonstrated over a 10% increase in knee extension moment at peak stress, and the peak knee extension moment during the cushioning phase was also significantly higher. These findings suggest that the elevated quadriceps force at peak stress may primarily be attributed to the increased knee extension moment, thereby contributing to a greater patellofemoral joint contact force. The observed changes in peak knee extension moment during the cushioning phase were inconsistent with findings from previous studies (Chen, Peng & Wei, 2022). These discrepancies may be attributed to differences in ASI material composition and structural support design. Future research should investigate how various combinations of material properties and structural configurations influence lower limb biomechanics.

In addition to transient loading on the patellofemoral joint, cumulative loading effects should also be considered. In the present study, the ASI group exhibited an increase of over 11% in the patellofemoral joint stress–time integral (I_PFJS_) during the cushioning phase compared to the OI group. Although elevated stress levels may contribute to the onset of pain (Zhang, Luo & Zhou, 2019), increased joint loading is not consistently associated with patellofemoral pain (Hart et al., 2022). Therefore, the causal relationship between elevated stress and symptom development remains inconclusive. Further research is warranted to determine whether long-term exposure to increased patellofemoral joint loading may directly contribute to symptom onset. Although current data do not prove that high stress necessarily causes symptoms, high stress may carry a higher risk of negative health outcomes for people with a history of knee problems (*e.g.*, athletes and enthusiasts who have suffered from patellofemoral pain). Therefore, caution is warranted. Overall, the findings indicate a biomechanical trade-off, in which external impact loading is reduced (as reflected by lower VLR), whereas patellofemoral joint stress is increased. It should be noted that the biomechanical effects of ASI may vary across different tasks or sport-specific movements.

### Limitations and prospects

The mathematical model employed in this study to estimate patellofemoral joint parameters was based on cadaveric data and was limited to the sagittal plane. Nevertheless, such models may not accurately capture individual variability or reflect the actual patellofemoral joint contact area during dynamic activities. As a result, the PFJS estimates from this model may not fully reflect the actual conditions in vivo. Future research could incorporate advanced imaging techniques, such as Dual Fluoroscopic Imaging System (DFIS) or Magnetic Resonance Imaging (MRI), to reconstruct patellofemoral joint contact areas using 3D inverse modeling for finite element analysis. Such 3D imaging techniques not only provide more accurate tracking of patellofemoral joint kinematics but also account for cartilage deformation, which could offer a more comprehensive representation of joint mechanics during dynamic activities. In addition, the level of support and cushioning provided by the selected badminton shoes and ASI may have influenced the results. Therefore, future studies should consider using multiple types of badminton shoe types in combination with different ASI designs to investigate potential interaction effects. Furthermore, future studies should include female subjects and examine biomechanical changes resulting from the body’s long-term adaptation to ASI, and explore the effects of individualized or customized ASI designs. Although the influence of sex differences on the biomechanical effects of ASI has not yet been established, such differences have been demonstrated in biomechanical studies of the badminton lunge. It should also be noted that the results of this study received the limitations of a relatively small sample size and the fact that only a single movement pattern was studied. Therefore, extrapolation of these results to other populations or footwork should be done with caution. In the future, there is a need to expand the sample size as well as to select a wider range of badminton sport-related movements. To strengthen the evidence, future research could incorporate multi-center study designs or randomized controlled trials for more generalizable results.

## Conclusions

In this exploratory study, we found that arch support insoles may reduce impact injury and increase patellofemoral loading during the landing phase of a badminton forward lunge through two mechanisms. Specifically, changing the ankle cushioning strategy reduces the average loading rate of the vertical ground reaction force and peak negative ankle power. It also reduces the occurrence of the ACL dangerous movement pattern associated with dynamic knee valgus. However, this alteration may lead to an increase in the extension moment, resulting in increased patellofemoral loading. It is important to note that this increase in load does not necessarily indicate the emergence of symptoms. Furthermore, these findings should be interpreted with caution, as the results of this study are preliminary and only apply to short-term testing, and cannot be generalized to long-term adaptive changes from prolonged use. Overall, the use of ASI in badminton should be approached with caution, considering both its potential positive biomechanical changes and the possible risk of increased joint loading. For coaches and sports medicine practitioners, ASI should be prescribed on a case-by-case basis to ensure that their mechanical effects align with the athlete’s specific needs and risk profile. Further exploration of insole material optimization and individualized fit strategies is needed to clarify the effects of ASI intervention on the patellofemoral joint.

## Supplemental Information

10.7717/peerj.21113/supp-1 Supplemental Information 1 Flowchart for calculation of patellofemoral joint stress The flowchart of the patellofemoral joint model. Created in part with reference to this article: Nunes GS, Scattone SR, dos Santos A, Fernandes R. 2018. Methods to assess patellofemoral joint stress: A systematic review. *Gait & Posture* 61:188–196. DOI: 10.1016/j.gaitpost.2017.12.018.

10.7717/peerj.21113/supp-2 Supplemental Information 2 Raw data

## Additional Information and Declarations

### Data Availability

The following information was supplied regarding data availability: The raw data is available in the Supplementary File.
