# Supplementary figures and images for: The immediate effect of arch support insoles on the biomechanics of the lower limbs during the forward lunge step in badminton
Source: PeerJ. 2026 Apr 14;14:e21113. doi: 10.7717/peerj.21113 (PMC13089218; doi:10.7717/peerj.21113)

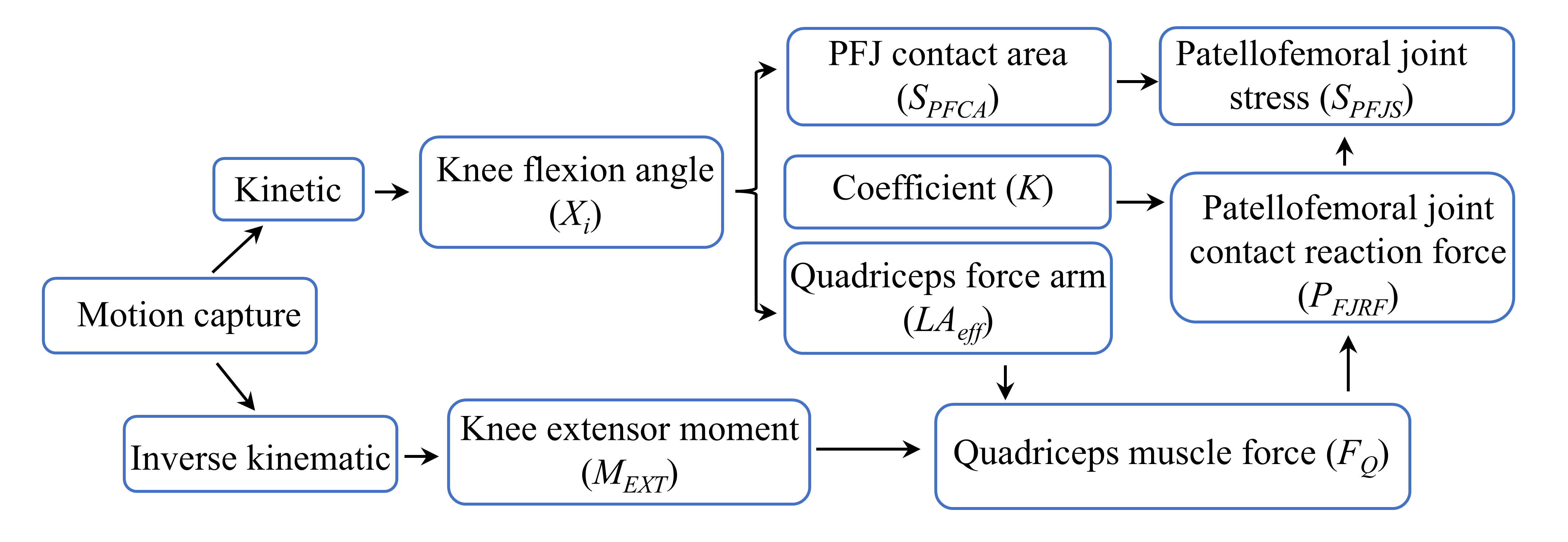

Supplement: Supplemental Information 1 — The flowchart of the patellofemoral joint model. Created in part with reference to this article: Nunes GS, Scattone SR, dos Santos A, Fernandes R. 2018. Methods to assess patellofemoral joint stress: A systematic review. Gait & Posture 61:188–196. DOI: 10.1016/j.gaitpost.2017.12.018. [file peerj-14-21113-s001.png]
